# Семейный случай гипогонадотропного гипогонадизма как проявление синдрома CHARGE

**DOI:** 10.14341/probl12748

**Published:** 2021-05-07

**Authors:** Д. А. Хабибуллина, Н. Ю. Калинченко, С. В. Егорова, Е. В. Васильев, В. М. Петров, А. Н. Тюльпаков

**Affiliations:** Национальный медицинский исследовательский центр эндокринологии; Национальный медицинский исследовательский центр эндокринологии; Детская краевая клиническая больница имени А.К. Пиотровича; Национальный медицинский исследовательский центр эндокринологии; Национальный медицинский исследовательский центр эндокринологии; Медико-генетический научный центр имени академика Н.П. Бочкова

**Keywords:** гипогонадотропный гипогонадизм, колобома, CHARGE-синдром, задержка полового развития, аномалии развития уха, ген <i>CHD7</i>, клинический случай

## Abstract

Синдром CHARGE — это редкое аутосомно-доминантное заболевание, обусловленное патологическими изменениями в гене CHD7. Особенностью данного синдрома является выраженный клинический полиморфизм даже среди носителей идентичной мутации. Спектр клинических проявлений варьирует от изолированной задержки полового созревания без необходимости в гормональной заместительной терапии до тяжелых множественных полиорганных пороков развития, требующих мультидисциплинарного терапевтического подхода. Диагноз можно заподозрить на основании сочетания главных и второстепенных критериев, однако окончательная верификация диагноза требует молекулярно-генетического исследования. Точная диагностика необходима не только для выбора корректной тактики ведения пациента, но и для информирования пациентов относительно всех возможных клинических проявлений заболевания, включая репродуктивный потенциал и риски наследования заболевания. Наиболее частым эндокринным отклонением при синдроме CHARGE является изменение гонадотропной функции от позднего пубертата до необратимого вторичного гипогонадотропного гипогонадизма, сочетающегося с нарушением обоняния различной степени выраженности, что обусловлено особенностями экспрессии гена CHD7 в эмбриональном периоде.

В статье представлено клиническое описание семейного варианта синдрома со значимой внутрисемейной вариабельностью клинических проявлений вследствие мутации в гене CHD7.

## Abstract (en)

CHARGE syndrome is a rare autosomal dominant disease caused by CHD7 gene mutations. Individuals with CHARGE display a wide spectrum of clinical features. It might be presented only as a delay puberty, which does not require any hormone replacement therapy to severe CHARGE phenotype, requiring a multidisciplinary therapeutic approach. Wild spectrum of clinical presentation can be seen even among the patients with identical mutation. Diagnosis might be suspected by a combination of major and minor clinical criteria of this disorder, but molecular genetic analysis is mandatory for final verification. Accurate diagnosis is essential to informing patients about all possible clinical features, reproductive status and choosing the correct treatment approach. The most common endocrine abnormality in patients with CHARGE syndrome is the disturbance in gonadotropins function ranged from delay puberty to persistent hypogonadotropic hypogonadism with different olfactory phenotypes, resulted by specific role of CHD7 in GnRH neuronal embryogenesis.

We describe a familial case of CHARGE syndrome with significant intrafamilial clinical heterogeneity due to CHD7 gene mutation.

## АКТУАЛЬНОСТЬ

CHARGE-синдром (OMIM no. 214800) представляет собой редкое генетическое заболевание с аутосомно-доминантным типом наследования, в основе которого лежат мутации в гене CHD7. Частота заболеваемости колеблется от 1:12 000 до 1:15 000 новорожденных [1][2].

Термин «CHARGE» — это аббревиатура, описывающая совокупность наиболее часто встречающихся клинических признаков, включающая колобому (Сoloboma), пороки сердца (Heart defects), атрезию/стеноз хоан (Сhoanal atresia), задержку роста и/или развития (Retardation of growth and/or development), пороки развития мочеполовой системы (Genitourinary Malformation) и аномалии развития уха (Ear abnormalities).

Первые клинические описания встречаются в литературе с 1979 г., когда Hitter и соавт. и Hall независимо друг от друга описали фенотипические особенности, присущие синдрому. В 1981 г. Pagon et al. [3] предложили объединить основные клинические проявления в аббревиатуру. В настоящий момент синдром CHARGE считается клиническим диагнозом, основанным на сочетании главных и второстепенных критериев, предложенных Blake и соавт. в 1998 г., а чуть позже расширенных и модифицированных Verloes и соавт. в 2005 г. [4]. Авторами также предложено выделять типичные/атипичные и парциальные формы синдрома (табл. 1).

**Table:** Таблица 1. Диагностические критерии синдрома CHARGE

Критерии Blake (1998)	Критерии Verloes (2005)	Модифицированные критерии Hale et al. (2016)
Диагноз сомнений не вызывает: 4 больших критерияили 3 больших + 3 малых (второстепенных).Диагноз возможен: 1–2 больших критерия и несколько второстепенных	Типичная форма: 3 больших критерияили 2 больших + 2 малых.Атипичная форма: 2 больших критерия при отсутствии малых или 1 большой + 2 малых.Парциальная форма:2 больших + 1 малый	CHARGE: 2 больших + несколько малых
БОЛЬШИЕ КРИТЕРИИ
•Колобома.•Атрезия/стеноз хоан.•Гипо- или аплазия полуокружных каналов внутреннего уха.•Дисфункция черепных нервов (преимущественно VII и VIII пары)	•Колобома.•Атрезия/стеноз хоан.•Гипо- или аплазия полуокружных каналов внутреннего уха	•Колобома.•Атрезия/стеноз хоан.•Аномалии уха, включая гипо- или аплазию полуокружных каналов.•Патогенные варианты гена CHD7
МАЛЫЕ КРИТЕРИИ
•Гипоплазия половых органов.•Задержка развития.•Дефицит роста.•Сердечно-сосудистые мальформации.•Пороки лицевого черепа.•Трахео-пищеводная фистула	•Дисфункция ромбоэнцефалических структур мозга (ствол/ черепные нервы).•Дисфункция гипоталамо-гипофизарной оси.•Мальформации внутреннего/наружного уха.•Аномалии внутренних органов (сердце, пищевод).•Расстройства интеллекта	•Дисфункция черепных нервов.•Структурные аномалии головного мозга.•Задержка развития/ расстройства интеллекта.•Дисфункция гипоталамо-гипофизарной оси.•Аномалии половых и внутренних органов (сердце, пищевод, почки)

В 2004 г. Vissers L.E. и соавт. [5] идентифицировали у пациентов с синдромом CHARGE патологические изменения в гетерозиготном состоянии в гене CHD7 (OMIM no. 608892), установив, таким образом, молекулярно-генетическую основу заболевания.

Благодаря возможности молекулярно-генетической верификации диагноза в литературе все чаще появляются описания синдрома CHARGE, ассоциированного с мутациями в гене CHD7, но без полной клинической картины заболевания, или так называемые атипичные мягкие фенотипы. Более того, отмечена значительная фенотипическая вариабельность даже среди членов одной семьи, имеющих одну и ту же патологическую замену. В связи с чем, учитывая аутосомно-доминантный тип наследования, наличие патогенного варианта CHD7 у членов одной семьи в сочетании с одним из больших диагностических критериев рекомендовано считать достаточным для подтверждения синдрома CHARGE, что позволяет поставить диагноз пациентам с атипичной или парциальной формой патологии [1][5].

В отечественной литературе описания синдрома CHARGE встречаются крайне редко, с преимущественным акцентом на тактику своевременных хирургических и терапевтических методов лечения врожденных пороков развития, начиная с раннего неонатального периода [6][7].

В статье представлено клиническое описание семейного варианта синдрома со значимой внутрисемейной вариабельностью клинических проявлений — от мягкой формы, характеризующейся изолированным нарушением полового созревания, до типичной формы с полным спектром клинических проявлений в результате дефекта гена CHD7.

## ОПИСАНИЕ СЛУЧАЯ

Пациентка А. впервые поступила в детское отделение ФГБУ «НМИЦ эндокринологии» Минздрава России в возрасте 14 лет с жалобами на задержку полового развития, низкие темпы роста, аносмию. По месту жительства проведено кариотипирование — кариотип 46,XX. Ребенок от 2-й нормально протекавшей беременности, 2-х самостоятельных срочных родов. При рождении длина тела 54 см, вес 3270 г. Из анамнеза известно, что брак родителей не близкородственный, наследственность отягощена: у отца — гипоосмия, задержка полового развития в анамнезе (в возрасте 25 лет получал терапию хорионическим гонадотропином (ХГЧ) и 3-месячный курс заместительной терапии препаратами эфиров тестостерона, после чего отмечено начало самостоятельного полового созревания), конечный рост — 180 см. У дяди по отцовской линии — два бесплодных брака. У родного брата пробанда — врожденная колобома, левосторонняя микрофтальмия, подковообразная почка, в возрасте 14 лет получал комбинированное лечение по поводу герминомы левого яичка, с 17 лет — гормональная заместительная терапия препаратами эфиров тестостерона. Мать — рост 160 см, менархе в 12,5 года (рис. 1). С рождения пациентка наблюдается в МНТК «Микрохирургия глаза» по поводу хориоретинальной центральной колобомы глаза, захватывающей диск зрительного нерва (ДЗН), врожденного микрофтальма правого глаза.

**Figure:**
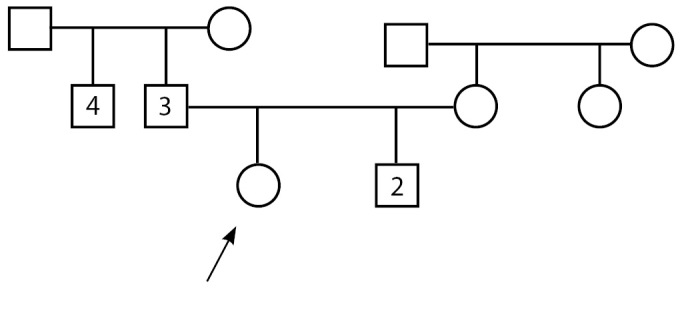
Рисунок 1.Примечание: у родного брата пробанда (2) — врожденная колобома, левосторонняя микрофтальмия, подковообразная почка, в анамнезе комбинированное лечение по поводу герминомы левого яичка, с 17 лет — гормональная заместительная терапия препаратами эфира тестостерона; у отца (3) — гипоосмия, задержка полового развития в анамнезе; у дяди по отцовской линии (4) — два бесплодных брака (не обследован).

При обследовании: рост 152 см (SDS роста: -1,47), вес 46,7 кг (SDS индекса массы тела: 0,2), обращал на себя внимание фенотип — колобома, микрофтальмия правого глаза, сходящееся косоглазие. Половое развитие по Таннеру (В 1, Р 1) Ах отсутствует, Ме abs.

В гормональном профиле отмечались допубертатные базальные значения половых гормонов: лютеинизирующий гормон (ЛГ) — 0,2 Ед/л (2,6–12,1), фолликулостимулирующий гормон (ФСГ) — 0,66 Ед/л (1,9–11,7), эстрадиол — 53,4 пмоль/л (97–592), антимюллеров гормон (АМГ)  — 2,8 нг/мл (0,1–9,85), ингибин В — 18,7 пг/мл (0–273), пролактин — 73,6 мЕд/л. Для уточнения характера задержки полового развития проведена проба с аналогом гонадотропин-рилизинг-гормона (ГнРГ), по результатам которой максимальный выброс ЛГ составил 0,9 Ед/л, что свидетельствует в пользу вторичного генеза гипогонадизма (табл. 2). Однако, учитывая семейный анамнез, не исключено более позднее начало самостоятельного полового развития.

**Table:** Таблица 2. Гормональный профиль пациентки до пробы с аналогом гонадотропин-рилизинг-гормона и по ее результатам

	ЛГ, Ед/л	ФСГ, Ед/л
Базальный уровень	0,2	0,66
Через 60 мин*	0,7	1,82
Через 240 мин	0,9	3,16

УЗИ органов малого таза: размеры матки и яичников соответствовали возрастной группе 2–7 лет. На рентгенограмме кистей отмечалась значимая задержка костного возраста (КВ соответствовал 11,5 годам по атласу TW20). При проведении МРТ головного мозга выявлены гипоплазия обонятельной луковицы справа и аплазия обонятельной луковицы слева. Учитывая отягощенный семейный анамнез по задержке полового развития, проведено молекулярно-генетическое исследование — секвенирование нового поколения с использованием панели «Гипогонадотропный гипогонадизм», где было выявлено наличие гетерозиготного варианта c.6193C>T:p.R2065C в гене CHD7 (NM_017780.4), описанного ранее в литературе при синдроме Кальмана (СК) [6]. Аналогичная замена обнаружена у отца и брата пробанда. На основании клинической картины, данных наследственного анамнеза, низких базальных показателей гонадотропинов и эстрадиола, данных пробы с аналогом ГнРГ в сочетании с аносмией и гипоплазией обонятельных луковиц по данным МРТ установлен диагноз: CHARGE-синдром: колобома, аносмия, гипогонадотропный гипогонадизм. В связи с чем был проведен скрининг на наличие других возможных компонентов заболевания: данных за наличие патологии сердца, органов мочевыделительной системы, хоан, органа слуха не получено.

Учитывая значимое отставание костного возраста, а также наличие генетически подтвержденного заболевания, с целью улучшения ростового прогноза и социальной адаптации пациентке была рекомендована заместительная гормональная терапия препаратами эстрадиола гемигидрата в минимальных стартовых дозах.

## ОБСУЖДЕНИЕ

CHARGE-синдром — патологическое состояние, обусловленное мутациями гена CHD7 (Chromodomain Helicase DNA binding protein 7), также известного как ДНК-связывающий белок 7 хромодоменной хеликазы. Ген локализован на длинном плече 8-й хромосомы (8q12) и состоит из 38 экзонов, первый из которых является некодирующим.

В течение первых 22 дней эмбрионального развития CHD7 экспрессируется повсеместно, регулируя транскрипцию ряда тканеспецифичных генов-мишеней, эффекты которых зависят от типа ткани и стадии развития. Поскольку максимальная экспрессия гена отмечена в недифференцированном нейроэпителии и мезенхиме нервного гребня, многие особенности синдрома можно отнести к нарушению процесса миграции клеток последнего [8].

На сегодняшний день описано более 500 патогенных вариантов CHD7. Наиболее распространенными являются нонсенс-мутации, вторыми по частоте — мутации со сдвигом рамки считывания, значительно реже встречаются сплайсинг-мутации. Миссенс-мутации чаще выявляются при изолированном варианте гипогонадотропного гипогонадизма и ассоциированы с более мягкими фенотипами [9]. При сдвиге рамки считывания или нонсенс-мутациях, приводящих к формированию функционально неактивного или аномального белка, чаще отмечается более тяжелое течение заболевания с вовлечением в патологический процесс более чем одного органа. Однако точной корреляции между типом мутации и степенью выраженности клинических проявлений сопутствующих дефектов даже среди пациентов с идентичными мутациями не отмечено [2][10–12].

Наиболее частой эндокринной аномалией, обнаруживаемой при синдроме CHARGE, является гипогонадотропный гипогонадизм (60–80% случаев). Клинические проявления изменений гонадотропной функции могут варьировать от позднего пубертата до стойкого вторичного гипогонадизма. У мальчиков гипогонадизм может быть заподозрен при наличии микропениса при рождении, чаще в сочетании с крипторхизмом. У девочек диагноз может быть заподозрен только в период пубертата, при отсутствии спонтанного полового созревания.

Частое сочетание гипогонадотропного гипогонадизма и аносмии, обусловленное нарушением миграции нейронов ГнРГ, является хорошо известным признаком СК. Поскольку CHD7 участвует в регуляции экспрессии генов во время эмбрионального развития, не исключено его влияние на действие или экспрессию ANOS1, FGFR1, FGF8, PROK2 и PROKR2 — генов, патологические изменения которых сопряжены с клиническими проявлениями СК. Поскольку СК является одним из фенотипов, наблюдаемых при CHARGE, авторы рекомендуют проводить скрининг CHD7 у пациентов с гипогонадизмом и аносмией, диагноз которых не был подтвержден молекулярно-генетически [8][9][13].

К настоящему моменту в литературе имеется упоминание двух случаев гипогонадотропного гипогонадизма, обусловленного гетерозиготным вариантом c.6193C>T:p.R2065C в гене CHD7 (NM_017780.4). У обоих описанных пациентов отсутствовал полный фенотип синдрома CHARGE. В обоих случаях представлены описания задержки полового развития с нарушением обоняния разной степени выраженности, в сочетании с фенотипическими особенностями, ассоциированными с атипичной и парциальной формой синдрома CHARGE (врожденный порок сердца, лицевого скелета и слуха), а также с упоминаниями о задержке полового развития у членов семьи пробандов. Таким образом, патогенность мутации не вызывает сомнений, подтверждая при этом литературные данные о значимом полиморфизме клинических проявлений [9][14].

## ЗАКЛЮЧЕНИЕ

Молекулярно-генетическое подтверждение диагноза важно для генетического консультирования и информирования пациентов относительно всех возможных клинических проявлений заболевания, в частности о потенциальных репродуктивных возможностях (в ряде случаев возможна более поздняя самостоятельная активация гипоталамо-гипофизарной оси, что не требует гормональной заместительной терапии или ее кратковременного назначения), а также рисках передачи заболевания своему потомству.
